# Early cardiotoxicity of radiotherapy in breast cancer patients after chemotherapy: the early warning sign

**DOI:** 10.1186/s43046-026-00341-2

**Published:** 2026-04-27

**Authors:** Zakka Zayd Zhullatullah Jayadisastra, Muhammad Ivan Aulia Sani, Lucia Kris Dinarti, Hasanah Mumpuni, Susanna Hilda Hutajulu, Wigati Dhamiyati

**Affiliations:** 1 Department of Cardiology and Vascular Medicine, Faculty of Medicine, Public Health, and Nursing, Gadjah Mada University, Yogyakarta, Indonesia; 2 Department of Internal Medicine, Faculty of Medicine, Public Health, and Nursing, Gadjah Mada University, Yogyakarta, Indonesia; 3 Hematology and Medical Oncology Division, Department of Internal Medicine, Faculty of Medicine, Public Health, and Nursing, Gadjah Mada University, Yogyakarta, Indonesia; 4 Division of Radiation Oncology, Department of Radiology, Faculty of Medicine, Public Health, and Nursing, Gadjah Mada University, Yogyakarta, Indonesia

**Keywords:** Radiotherapy, Chemotherapy, Cardiotoxicity

## Abstract

**Background and aims:**

Radiotherapy, like chemotherapy, in breast cancer can cause cardiotoxicity. Research about the combined effect of radiotherapy and chemotherapy on left ventricular diastolic function (LVDD) is limited. Therefore, this study was conducted to see the early effect of radiotherapy in patients after completing chemotherapy.

**Method:**

This research was a prospective analytical observational study with a pre-post design that was conducted from August 2023 to August 2024 in breast cancer patients who had completed their chemotherapy in Sardjito Hospital. The primary endpoint was cardiotoxicity in any level according to European Society of Cardiology (ESC) 2022 cardio-oncology guideline. Cardiotoxicity was diagnosed by considering patients’ history, physical examination, and echocardiography results.

**Result:**

Twenty-nine female subjects were enrolled. Seven subjects (24.14%) had cardiotoxicity with one of them was symptomatic. McNemar test showed significant effect of radiotherapy (*p* = 0.016). Types of chemotherapy were not a confounding factor in this study.

**Conclusion:**

Radiotherapy may cause mild cardiotoxicity in short term. A routine screening protocol is needed to prevent further complication.

**Supplementary Information:**

The online version contains supplementary material available at 10.1186/s43046-026-00341-2.

## Introduction

Breast cancer is a commonly diagnosed cancer and the commonest cause of death due to cancer in women worldwide. Metastatic breast cancer, which could spread by the vascular or lymphatic system, has a worse outcome in these patients. Early diagnosis and treatment increase the life expectancy in breast cancer [1–3]. Standard therapies that are available are chemotherapy, radiotherapy, and surgery. Therapy modalities are chosen by matching them with the type and stage of the cancer. The main goal in breast cancer therapy is destroying the cancer cell and containing the local cancer malignancy [4, 5].

Radiotherapy, like chemotherapy, could disturb cardiac function, commonly called cardiotoxicity. Cardiotoxicity of radiotherapy could emerge acutely or chronically. Disturbances that could be manifested are systolic dysfunction, diastolic dysfunction, conduction disturbances, and pericardial diseases. Left ventricular diastolic dysfunction (LVDD) emerges the earliest in the patient, as fast as three months after radiotherapy. Some factors that supported cardiotoxicity in radiotherapy are cumulative dose more than 30 Gy, mean heart dose (MHD) more than 3 Gy, younger patients, traditional cardiovascular risk, cardiotoxic chemotherapy, and longer time after radiotherapy [6–11]. Interestingly, there were no significant differences in cardiotoxicity, especially left ventricular diastolic dysfunction, between left and right breast cancer [12].

The National Comprehensive Cancer Network (NCCN) suggested radiotherapy to be done after surgery or chemotherapy [13]. This made research about cardiotoxicity after radiotherapy without chemotherapy difficult to conduct. Studies about the combination effect of chemotherapy and radiotherapy in left ventricular diastolic dysfunction were also limited. This study was trying to fill this gap to see the early effect of radiotherapy on cardiotoxicity in breast cancer patients after chemotherapy.

## Method

This is a prospective observational study with a pretest-posttest design in one group without control. This study was conducted from August 2023 to August 2024. Clinical data and echocardiography parameters were taken from Sardjito Hospital patients that were going to undergo radiotherapy. The target population of this research was patients with breast cancer in stages I-III that had completed a chemotherapy cycle and would undergo radiotherapy. Samples were taken with a consecutive sampling method that satisfied the inclusion and exclusion criteria.

Inclusion criteria were being a minimum of 18 years old, female, diagnosed with breast cancer stage I-III, having completed an echocardiography examination, having completed a chemotherapy cycle, and being indicated for radiotherapy according to protocol at Sardjito Hospital. Exclusion criteria were having technical difficulties in data acquirement (e.g., lung tuberculosis, a wound in the left chest area, or difficulty in presenting at the study site at the designated time) and patients with a history of coronary artery disease, systolic dysfunction, atrial fibrillation, atrial flutter, pulmonary hypertension, or any other diseases that made the patient unable to undergo radiotherapy (e.g., anemia, sepsis, or pregnancy) before radiotherapy. Drop-out criteria were the patient being unable to complete the radiotherapy protocol and the patient not undergoing an echocardiography examination in the designated time.

The patients that were enrolled in the study then underwent radiotherapy and were called for clinical and echocardiography examination three months following the completion of radiotherapy. The data then was acquired and analyzed.

The independent variable was radiotherapy. Dependent variables were cardiotoxicity. Confounding variables were radiation dose, age, chemotherapy regimen, hypertension, diabetes mellitus, dyslipidemia, history of smoking, and obesity.

Radiotherapy was defined as one cycle of radiotherapy according to Sardjito Hospital protocol that was adapted from NCCN guideline 13 with planning of IMRT (intensity-modulated radiotherapy) or 3D CRT (three-dimensional conformal radiotherapy) as they were available. Radiation dose and planning were taken from the workstation of the radiotherapy. Diastolic function was a diastolic parameter that was acquired and analyzed using echocardiography according to American Society of Echocardiography recommendation [14]. Transthoracic echocardiography was done in patients lying down and oblique to the left. All parameters were taken twice and averaged. GLS (Global longitudinal strain) was taken using the software of the machine. E (early maximum mitral diastolic flow velocity) and A (late maximum mitral diastolic flow velocity) were measured with pulsed wave Doppler. Septal and lateral e’ (early diastolic mitral annular velocity) were measured with tissue Doppler. M-mode view was used to measure left ventricular end diastolic diameter and left ventricular end systolic diameter. Apical four-chamber view and apical two-chamber view were used to measure left atrial volume index. Continuous wave Doppler was used to measure the maximum regurgitation velocity of the tricuspid valve. The machines used were GE^®^ Vivid T8 or GE^®^ Vivid E95. Clinical data (age, chemotherapy regimen, hypertension, diabetes mellitus, dyslipidemia, history of smoking, and obesity) were taken from medical records.

The primary endpoint was cardiotoxicity in any level according to European Society of Cardiology (ESC) 2022 cardio-oncology guideline (Table 1). Cardiotoxicity was diagnosed by considering patients’ history, physical examination, and echocardiography results. Biomarkers were not taken in this research due to limited resources.

**Table 1:** Cancer therapy-related cardiovascular toxicity definitions (Reproduced from [15])

Symptomatic CTRCD	Very severe	HF requiring inotropic support, mechanical circulatory support, or consideration of transplantation
	Severe	HF hospitalization
	Moderate	Need for outpatient intensification of diuretic and HF therapy
	Mild	Mild HF symptoms no intensification of therapy required
	Very severe	HF requiring inotropic support mechanical circulatory support, or consideration of transplantation
Asymptomatic CTRCD	Severe	New LVEF reduction to < 40%
	Moderate	New LVEF reduction by ≥ 10% points to an LVEF of 40–49% *OR* New LVEF reduction by < 10% points to an LVEF of 40–49% AND either new relative decline in GLS by > 15% from baseline OR new rise in cardiac biomarkersc
	Mild	LVEF ≥ ***50%*** AND new relative decline in GLS by > 15% from baseline AND/OR new rise in cardiac biomarkers

*Abbreviation* *LVEF* Left ventricular ejection fraction, *GLS* Global longitudinal strain, *HF* Heart Failure

**Table 2:** Baseline Characteristics of the Study (N = 29)

Characteristics	Data (*n*(%))
Age (years old)*	50.6 ± 7.4
Breast Cancer Site	
Right	17(58.62)
Left	12(41.38)
Radiotherapy Planning	
3D CRT	12(41.38)
IMRT	17(58.62)
Radiation Dose (Gy)*	52.38 ± 3.62
Mean Heart Dose (Gy)*	1.09 ± 0.44
Overall Treatment Time (days)*	59.2 ± 13.8
Chemotherapy Used	
Anthracycline	20(68.96)
Anthracycline Cumulative Dose	1092.65 ± 443.92
Anti CDK 4/6	0(0)
Anti HER−2	3(10.34)
5-Fluorouacil	2(6.90)
Cyclophosphamide	18(62.07)
Carboplatin	7(24.14)
Taxane	12(41.38)
Bortezomib	1(3.44)
Comorbidities	
Hypertension	6(20.69)
Dyslipidemia	1(3.45)
Diabetes mellitus	0(0)
Obesity	2(6.89)
Smoking	0(0)
Blood Pressure (mmHg)	
Systole*	119 ± 13
Diastole*	72 ± 9
Heart rate (bpm)*	90 ± 13

*Expressed in mean ± SD

*Abbreviations* *SD* Standard deviation, *bpm* beat per minute, *mmHg* milimeter mercury, *Gy* Gray, *3D CRT* three-dimensional conformal radiotherapy, *Anti CDK 4/6* Anti cyclin dependent kinase 4/6, *Anti HER−2* Anti human epidermal growth factor−2, *IMRT* Forward-planned intensity-modulated radiotherapy

Statistical analysis was conducted in IBM^®^ SPSS Statistics Version 26. The McNemar test was used to see the effect before and after radiotherapy. Subanalysis of numerical variables from diastolic function was done using either a paired T-test or a Wilcoxon signed-rank test, whichever was feasible. *P* < 0.05 was considered statistically significant. If the result were significant, multivariate logistic analysis would be conducted to see the risk factor of the result. A confounding variable that has *p* < 0.01 would be considered significant to be a risk factor. Non parametric single group binomial test was used to see the difference of chance in different cardiotoxicity stratification.

The minimum study size that we expected was calculated using the formula for a two-tailed McNemar study with an alpha of 0.05 and a confidence power of 80% [16] as written below:

*n* = 16pq(1-r)/d2.

n = minimum sample needed.

p = p1 + p2.

q = 1-p.

p1 = proportion of exposed sample with outcome.

p2 = proportion of unexposed sample with outcome.

r = phi correlation coefficient, assumed that there was no correlation to maximize the sample size (0).

d = |p2 - p1|.

Proportion of exposed sample with outcome (p1) were taken from Cao et al. [12] which was 39.7%, and proportion of unexposed sample with outcome (p2) was decided 0%. Therefore, we estimated the minimum sample size of 23 subjects.

## Result

**Fig. 1:**
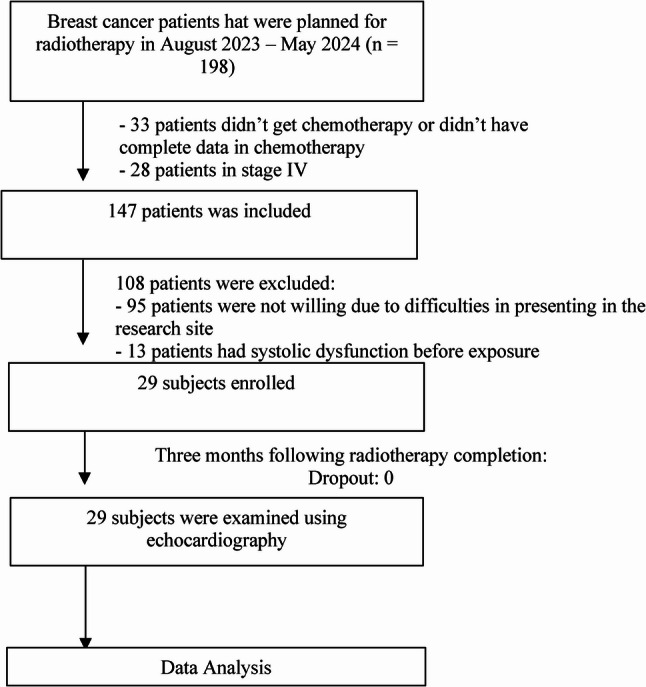
Study subject recruitment and study flow

198 patients were potentially enrolled in this study. 60 patients were not included because of not undergoing chemotherapy or incomplete data in chemotherapy (33 patients) or stage IV of breast cancer (28 patients). 95 patients were excluded due to inability to present at the examination site, and 13 patients were excluded due to having prior systolic dysfunction. 29 subjects were enrolled in the end without any dropouts in this study (Fig. 1).

Baseline characteristics of the study could be found in Table 2. The average age was 50.6 ± 7 years old, with all of the sex being female. 20.69% had a history of hypertension, 3.45% had a history of dyslipidemia, and 6.86% had obesity. No subjects had a history of diabetes mellitus and smoking.

The majority of the subjects had right-sided breast cancer (58.62%). The commonest chemotherapy regimen was anthracycline (68.96%) and the commonest radiotherapy planning used was IMRT (58.62%). The average total dose and mean heart dose used were 52.38 ± 3.62 Gy and 1.09 ± 0.44 Gy, respectively, and the average overall treatment time (OTT) was 59.2 ± 13.8 days. Average blood pressure was 119 ± 13/72 ± 9 mmHg, and average heart rate was 90 ± 13 bpm.

Seven of 29 subjects had cardiotoxicity after 3 months following radiotherapy. Six of seven subjects with cardiotoxicity were asymptomatic and had LVEF ≥ 50% and new relative decline in GLS by > 15% from baseline, thus classified as the asymptomatic mild cardiotoxicity. One of them was symptomatic with mild cardiotoxicity. The normality of the data was not tested due to the paired dichotomous logistic type of the data. McNemar statistical analysis exposed significant difference before and after radiation therapy (*p* = 0.016). Bivariate analysis comparing subjects that have cardiotoxicity showed no significant differences between confounding factors (Suppl 1).

The subject with symptomatic moderate cardiotoxicity was 63 years old and underwent radiotherapy with IMRT planning with a total dose of 50 Gy and a mean heart dose of 0.789 Gy in 38 days. The subject had a history of hypertension with blood pressure of 114/101. She also had a heart rate of 98 bpm and a body mass index (BMI) of 23.42 kg/m². The subject underwent Taxane chemotherapy. Echocardiography showed left ventricular diastolic dysfunction grade I (GLS−16.5%; E 42 cm/s; E/A 0.53; lateral e’ 5 cm/s; septal e’ 3 cm/s; E/e’ 10.5, no tricuspid regurgitation, and LAVI 22 mL/m2) with regional wall motion abnormality and eccentric hypertrophy of the left ventricle.

Subanalysis of diastolic function and detailed parameter analysis were conducted. Shapiro-Wilk analysis showed the normal distribution in mitral E, septal e’, and lateral e’. Statistical analysis showed significant difference in GLS (−21.9 ± 2.8 vs−19.7 ± 4.9; *p* = 0.012) but no significant differences in traditional diastolic parameters between pre- and post-radiotherapy (Table 3). Differences in parameters between subjects with symptomatic cardiotoxicity and the average of subjects without symptomatic cardiotoxicity were laid out in Table 4.

**Table 3:** Comparison of data before and three months following radiotherapy

	Before RT	Three months after RT	Mean difference	*p*
Cardiotoxicity ^&^	0 (0%)	7 (24.14%)	7 (24.14%)	0.016^@^
LVEF (%) ^$^	69.2 ± 7.1	70.3 ± 8.3	−1.14 ± 1.74	0.52
GLS (%) ^$^	−21.9 ± 2.8	−19.7 ± 3.3	2.17 ± 4.9	0.012^@^
E mitral (cm/sec) ^$^	77.07 ± 18.08	71.41 ± 20.79	5.66 ± 4.92	0.26
e’ septal (cm/sec) ^$^	9.45 ± 2.46	9.93 ± 3.14	−0.48 ± 0.75	0.52
e’ lateral (cm/sec) ^$^	13.48 ± 3.30	13.59 ± 0.61	−0.10 ± 0.58	0.86
E/e’ ^$^	6.98 ± 2.02	6.36 ± 1.70	0.62 ± 0.52	0.25
E/A^	1.04 ± 0.31	1.39 ± 1.81	−0.35 ± 0.35	0.14
TRV Max (m/sec)^	0.61 ± 1.01	0.70 ± 1.08	−0.1 ± 0.23	1
LAVI (mL/m2) ^$^	19.9 ± 5.01	21.76 ± 3.96	−1.86 ± 0.97	0.06
LVDD ^&^	0 (0%)	1 (3,45%)	1 (3,45%)	1

*Cardiotoxicity and LVDD are expressed in n(%). Other data is expressed in mean ± SD. ^**$**^Data was analyzed using paired T-Test. ^Data was analyzed using Wilcoxon Signed Rank Test. ^**&**^Data was analyzed using McNemar Test. ^@^Statistically significant

*Abbreviations* *GLS* Global longitudinal strain, *LVEF* Left ventricular ejection fraction, *SD* Standard deviation, *TRV Max* Tricuspid regurgitation velocity maximum, *LAVI* Left atrial volume index, *RT* Radiotherapy, *cm* centimeter, *m* meter, *mL* mililiter, *m2* meter square

**Table 4:** Comparison of echocardiography parameters three months following radiotherapy between subject with symptomatic cardiotoxicity and average of subjects without symptomatic cardiotoxicity

	Subject with symptomatic cardiotoxicity	Mean of samples without symptomatic cardiotoxicity	Difference
LVEF (%)	53	69.2 ± 7.1	−16
GLS (%)	−16.5	−19.8 ± 3.3	+ 3.3
E mitral (cm/sec)	42	77.07 ± 18.08	−35
e’ septal (cm/sec)	3	9.45 ± 2.46	−6.45
e’ lateral (cm/sec)	5	13.48 ± 3.30	−8.48
E/e’	10.5	6.98 ± 2.02	+ 3.52
E/A	0.53	1.04 ± 0.31	−0.51
TRV Max (m/sec)	0	0.61 ± 1.01	−0.061
LAVI (mL/m2)	22	19.9 ± 5.01	+ 2.1

Expressed in mean ± SD

*Abbreviations* *GLS* Global longitudinal strain, *LVEF* Left ventricular ejection fraction, *SD* Standard deviation, *TRV Max* Tricuspid regurgitation velocity maximum, *LAVI* Left atrial volume index, *RT* Radiotherapy, *cm* centimeter, *m* meter, *mL* mililiter, *m2* meter square

## Discussion

This is one of the pioneer studies of the combination effect of cardiotoxicity of chemotherapy and radiotherapy in subjects with breast cancer. Some prior studies that we found either used a concurrent single agent of chemotherapy while undergoing radiotherapy [16] or did not use chemotherapy at all [17–19].

The baseline characteristics of this study showed younger age than prior studies by Walker et al. [17] and Sritharan et al. [18]. The samples of the study also have lower prevalence of hypertension, dyslipidemia, and diabetes mellitus compared to the study from Walker et al. [17]. Mean blood pressure was also less than the previous study [17]. There were no prior studies that mentioned heart rate that we could compare our results with. The majority of the subjects had right-sided breast cancer, which is different from previous studies [12, 17]. Anthracycline was the most prevalent agent used in this study, different from any previous studies ever mentioned [12, 17–20].

The most prevalent radiotherapy planning used in this study was IMRT; this was similar to what was reported by Cao et al. [12], and the total dose was similar to Sritharan et al. [18]. There were limited studies that mentioned overall treatment time.

Cardiotoxicity was significant in three months following radiotherapy. This supports the finding from Cao et al. [12] and Sritharan et al. [18] but contradicts the result a bigger study by Berlin et al. [21]. The difference in this result might stems from the lack of cardiotoxicity identification and a lot more focus on the absolute numbers of the cardiac functions. Therefore, we proposed the comprehensive screening of cardiotoxicity with the diagnosis of cardiotoxicity based on published guideline to detect cardiotoxicity more precisely and to reduce morbidity and mortality due to cardiotoxicity [15].

We found a significant difference in GLS between pre- and post-radiotherapy. This supports the report from Walker et al. [17] that mentioned GLS difference in 6 weeks post-radiotherapy. Interestingly, there was no diastolic dysfunction according to traditional diastolic function measurement; this is different from what was reported by Cao et al. [12] in six months after radiotherapy, Walker et al. [17] also reported change in global longitudinal strain in six months after radiotherapy. However, this result was similar to Cao et al. [12] at the end of radiotherapy and three months after radiotherapy and also similar to Sritharan et al. [18], who reported no changes in traditional diastolic parameters at the time of radiotherapy and six weeks following radiotherapy.

Chemotherapy might not have role in cardiotoxicity in short term. Cao et al. [12] reported usage of trastuzumab, while our study reported majority usage of anthracycline. Anthracycline causes cardiotoxicity by producing reactive oxygen species that modify cardiolipin, which causes cytochrome C production, activation of p38 MAPK, and then induction of apoptosis in cardiomyocytes [22]. It also causes cardiotoxicity by creating a bond with iron ions that causes fat peroxidation and alteration of the mitoferrin transporter, which causes iron accumulation in mitochondria that causes cellular damage due to the free radical complex of anthracycline-iron [23]. Anthracycline also causes cardiomyocyte atrophy that leads to LV mass and function reduction [24]. These processes take five years after chemotherapy [25]. The combination effect with radiotherapy that was observed was one retrospective study in Hodgkin lymphoma that reported heart failure after 20 years in a cardiac dose of more than 20 Gy [26]. Interestingly, there were also non significant results of confounding factors, including regiment of chemotherapy. This showed that chemotherapy might not affecting cardiotoxicity in short term, although combined with radiotherapy. On the other hand, radiotherapy could single-handedly caused acute subclinical LVDD, which was shown in this research and in report by Sritharan et al. [18].

We tried to synthesize the result of the quantitative analysis of all samples and the qualitative analysis of one sample that had LVDD in this study. It is concluded that the result may be affected by the follow-up time point since there are many studies that reported LVDD according to traditional parameters after more than three months following radiotherapy [12, 19]. Although some studies suggested earlier LVDD, all of the studies reported it using more subclinical parameters [1, 19].

One sample that had symptomatic cardiotoxicity and LVDD had an older age compared to the average of samples that didn’t have LVDD (63 years old versus 50.6 years old). This may cause a decrease in diastolic function, especially for people over 60 years old [14]. Older people had lower early peak filling velocity (E), higher end filling velocity (A), and a lower ratio of E/A due to relative subendocardial dysfunction that causes lowering of cardiac output and end diastolic volume index [20]. On the other hand, this subject also had diastolic hypertension, which might also be caused by aging. Aging causes stiffness in systemic blood vessels that will lead to hypertension. Hypertension could increase systemic vascular resistance and then alter LV diastolic function [26, 27].

This is supported by the echocardiography data that showed lower E (42 m/sec versus 71.41 m/sec) and lower E/A (0.53 versus 1.39). This subject also had lower e’ lateral (5 cm/sec versus 9.93 cm/sec) and e’ septal (3 cm/sec versus 13.59 cm/sec) and higher E/e’ (10.5 versus 6.36) that showed lower left ventricular (LV) relaxation and restoring forces (myocardium movement into atrium) and higher LV filling pressure due to aging and regional wall motion abnormality in this subject [14, 28].

The taxane class of chemotherapy will increase reactive oxygen species and vascular stiffness. This effect could lead to systemic hypertension and reduction of longitudinal and area strain of the left ventricle that will end in LVDD [29]. Although Alghafar et al. [30] reported no significant cardiotoxicity difference between subjects with and without hypertension that used trastuzumab, Cao et al. [12] reported the opposite in his research that may be related to the combination with radiotherapy. This may be the underlying process in LVDD for the subject in this study.

Myocardial dysfunction due to radiotherapy was caused by formation of free radicals that contiued to molecular disturbances and tissue damages. Endothelial tissues changed into proinflammatory tissues that could damage blood vessels by oxidative stress, reactive oxygen species formation, and cytocines that could destroy integrity of deoxyribonucleic acid (DNA) [31–33]. This led to damage in vessel walls, thrombocyte aggregation, thrombosis, myofibroblast change and atherosclerotic disease [31–33] and restrictive cardiomyopathy and diastolic dysfunction caused by capillary problem that could lead into cell death, increase in proinflammatory cytocine, and increase of smooth muscle cell differentiation into myofibroblast and increase of collagen production [31, 34, 35].

Although the total dose of this study was higher than was recommended by NCCN [13] (52.38 ± 3.62 Gy versus 30–35 Gy), the mean heart dose is lower than the dose that increases the risk of cardiotoxicity from prior studies (1.09 ± 0.44 Gy versus 3 Gy); this may be the reason why radiotherapy didn’t cause LVDD in traditional parameters but only reducing GLS [6, 7].

The main strength of this study is its prospective design and its consecutive sample acquisition that ensures us to take as many subjects as possible. We also tried to incorporate the total radiation dose, mean heart dose, and overall treatment time to better understand the nature of cardiotoxicity in radiotherapy. One of the potential biases that we identified was the inability of many potential subjects to join the study due to multiple problems like economic, social, geographical, or private problems that were unable to be disclosed. Some potential subjects also did not accept the offer to be enrolled for being “a little unwell”. Therefore, a formal surveillance protocol should be established first in the future to get more subjects for future research.

There are several limitations to this study that need to be addressed. This is a pre-post observational study that only sees one point after exposure. It is better to evaluate at multiple time points to see the trend of the diastolic function and to better predict the cardiotoxic effect of radiotherapy and chemotherapy. Biomarker and cumulative dose of anthracycline were also not taken in this study due to resources limitation. This is a relatively small cohort in a single center that could be improved more in the future. Future directions of interest might include these analyses in larger cohorts at multiple time points, inclusion of other diastolic parameters, analysis of other potential cardiotoxicities, and more detailed data acquisition in radiation dosing, e.g. left ventricular and left artery descendant dose, which could allow more detailed analysis.

## Conclusion

In this prospective cohort study of women with breast cancer undergoing radiotherapy after chemotherapy we found significant number of mild cardiotoxicity in three months after radiotherapy, that was scarcely addressed in other studies. There is a need to further research in radiotherapy alone, without chemotherapy, to see its role in early cardiotoxicity without being influenced by other therapies. Although many were asymptomatic, this should be an enough wake up call for an established screening for patients undergoing radiotherapy.

## Supplementary Information

Supplementary Material 1.

## Data Availability

No datasets were generated or analysed during the current study.

## Abbreviations

3D CRT: Three-dimensional conformal radiotherapy
Anti CDK 4/6: Anti cyclin dependent kinase 4/6
Anti HER 2: Anti human epidermal growth factor−2
Gy: Gray
IMRT: Intensity-modulated radiotherapy
LV: Left ventricular
LVDD: Left centricular diastolic dysfunction
LVEF: Left ventricular ejection fraction
NCCN: National comprehensive cancer network
OTT: Overall treatment time
RT: Radiotherapy
TRV Max: Tricuspid regurgitation velocity maximum
VMAT: Volume-modulated arc therapy
