# Supplementary material for: Early cardiotoxicity of radiotherapy in breast cancer patients after chemotherapy: the early warning sign
Source: J Egypt Natl Canc Inst. 2026 Apr 27;38:13. doi: 10.1186/s43046-026-00341-2 (PMC13262374; doi:10.1186/s43046-026-00341-2)
Supplement: Supplementary file 1 — Supplementary Material 1. [file 43046_2026_341_MOESM1_ESM.docx]

Suppl 1. Bivariate analysis of cofounding factors

| **Confounding Factors** | | **Cardiotoxicity** | | | | **p** |
| --- | --- | --- | --- | --- | --- | --- |
|  |  | **No** | | **Yes** | |  |
|  |  | **n** | **%** | **n** | **%** |  |
| **Hypertension** | **Yes** | 17 | 73.9 | 6 | 26.1 | 0.999 |
|  | **No** | 4 | 66.7 | 2 | 33.3 |  |
| **Hormonal Therapy** | **Yes** | 16 | 76.2 | 5 | 23.8 | 0.646 |
|  | **No** | 5 | 62.5 | 3 | 37.5 |  |
| **Body Mass Index** | **<25 kg/m2** | 14 | 66.7 | 7 | 33.3 | 0.525 |
|  | **≥25 kg/m2** | 7 | 87.5 | 1 | 12.5 |  |
| **Radiotherapy Planning** | **3D CRT** | 9 | 75 | 3 | 25 | 0.999 |
|  | **IMRT** | 12 | 70.6 | 5 | 29.4 |  |
| **Anti HER-2** | **Yes** | 2 | 66.7 | 1 | 27.6 | 0.5 |
|  | **No** | 19 | 73.1 | 7 | 26.9 |  |
| **Age** | **<60 years old** | 18 | 75 | 6 | 25 | 0.597 |
|  | **≥60 years old** | 3 | 60 | 2 | 40 |  |
| **Overall Treatment Time** | **<40 days** | 1 | 33.3 | 2 | 66.7 | 0.176 |
|  | **≥40 days** | 20 | 76.9 | 6 | 23.1 |  |
| **Breast Cancer Location** | **Right** | 12 | 70.6 | 5 | 29.4 | 0.999 |
|  | **Left** | 9 | 75 | 3 | 25 |  |
| **Anthracycline** | **Yes** | 15 | 75 | 5 | 25 | 0.675 |
|  | **No** | 6 | 66.7 | 3 | 33.3 |  |
| **Mean Heart Dose** | **<1.92 Gy** | 9 | 56.3 | 7 | 43.8 | 0.483 |
|  | **≥1.92 Gy** | 1 | 92.3 | 2 | 7.7 |  |
| **Chemotherapy-Radiotherapy Interval** | **<12 weeks** | 16 | 76.2 | 5 | 23.8 | 0.646 |
|  | **≥12 weeks** | 5 | 62.5 | 3 | 37.5 |  |

Note: 3D CRT: three-dimensional conformal radiotherapy; Anti HER-2: Anti human epidermal growth factor-2. IMRT: Forward-planned intensity-modulated radiotherapy
